# *BMC Musculoskeletal Disorders* reviewer acknowledgement, 2015

**DOI:** 10.1186/s12891-016-0950-x

**Published:** 2016-02-24

**Authors:** James Mockridge

**Affiliations:** BioMed Central, Floor 6, 236 Gray′s Inn Road, London, WC1X 8HB UK

## Abstract

The editors of *BMC Musculoskeletal Disorders* would like to thank all our reviewers who have contributed to the journal in Volume 16 (2015).

**Ellen Aartun**

Canada

**H Abdelbary**

Canada

**Abdellatif Abdelgaied**

UK

**Ashraf Abdelkafy**

Egypt

**Adham Abdelrahim**

USA

**Christina Abdel Shaheed**

Australi

**Abhishek Abhishek**

UK

**Mansour Abolghasemian**

Iran

**Antonio Abramo**

Sweden

**Jonathan Adachi**

Canada

**Jo Adams**

UK

**Adewale Adebajo**

UK

**Babatunde Adegoke**

Nigeria

**Ade Fatai Adeniyi**

Nigeria

**Rintje Agricola**

Netherlands

**Lamiaa Ahmed**

Egypt

**Yong Ahn**

South Korea

**Yasushi Akamatsu**

Japan

**Hiroshi Akima**

Japan

**Toru Akiyama**

Japan

**Esin Aktas Cetin**

Turkey

**Ali Al Kaissi**

Austria

**Christoph Albers**

Switzerland

**John Albright**

USA

**Luis M. Alegre**

Spain

**Håkan Alfredson**

Sweden

**Hamza Alizai**

USA

**Saad Alsaadi**

Australia

**Tjarco D.W. Alta**

Netherlands

**Saad Altaher**

Saudi Arabia

**Rd Altman**

USA

**Norio Amizuka**

Japan

**Juan-Manuel Anaya**

Colombia

**Amy Anderson**

UK

**I. Andia**

Spain

**Dimosthenis Andreou**

Germany

**Jose Luis Andreu**

Spain

**Stephen Andrews**

Canada

**James Andrews**

USA

**Ricardo Angeles**

Canada

**Chiara Angeletti**

Italy

**Naffis Anjarwalla**

UK

**Narender Annapureddy**

USA

**Lieven Annemans**

Belgium

**Samuel Antwi-Baffour**

Ghana

**Daisuke Araki**

Japan

**Nigel Arden**

UK

**Luca Paolo Ardigò**

Italy

**Kristina Areskoug Josefsson**

Sweden

**Martin Aringer**

Germany

**Terra Arnason**

Canada

**Toni Arndt**

Sweden

**Camilla Arvinius**

Spain

**Elisa Assirelli**

Italy

**Diego Astur**

Brazil

**Henry Dushan Edward Atkinson**

UK

**Halil Atmaca**

Turkey

**Mukundan Attur**

USA

**Fabiola Atzeni**

Italy

**Angelo Gabriele Aulisa**

Italy

**Iben Axén**

Sweden

**Mostafa Ayoub**

Egypt

**Daniel Azevedo**

Brazil

**Debora Azevedo**

Brazil

**Fabio Azevedo**

Brazil

**Jaume Bacardit**

UK

**Frances Bach**

Netherlands

**Dawid Baczkowicz**

Poland

**Carlos Bagley**

USA

**Clemens Baier**

Germany

**Daniel Balbachevsky**

Brazil

**Antoine Baldassari**

USA

**Alejandro Balsa**

Spain

**Raveendhara Bannuru**

USA

**Mary Barbe**

USA

**Kim Barber Foss**

USA

**Alexandre Barbosa**

Brazil

**Thomas Bardin**

France

**William R. Barfield**

USA

**Alexej Barg**

Switzerland

**Antonio Barquet**

Uruguay

**Helena Barreto Henriksson**

Sweden

**Dominik Baschera**

Switzerland

**Caroline Bastiaenen**

Netherlands

**Marcus Bateman**

UK

**Christoph Bauer**

Switzerland

**Catherine Bauge**

France

**Petra Bäumler**

Germany

**Murad Bavbek**

Turkey

**Adam Baxter-Jones**

Canada

**Anne-Christine Bay-Jensen**

Denmark

**David Bazett-Jones**

USA

**Christoph Becher**

Germany

**Paula Regina Beckenkamp**

Australia

**Marc Beirer**

Germany

**Knut Beitzel**

Germany

**Bulent Bektaser**

Turkey

**Mary Bell**

Canada

**Adam Benham**

UK

**Kathleen Bennett**

Ireland

**Marco Bergamin**

Italy

**Mercedes G. Bermudez**

Spain

**Emilio Besada**

Norway

**Andrew Beswick**

UK

**Bryan Beutel**

USA

**Rahul Bhattacharyya**

UK

**Harjit Pal Bhattoa**

Hungary

**Roland Biber**

Germany

**Teresa Binkley**

USA

**Amelia J Birney**

USA

**Leanne Bisset**

Australia

**Emmanuel Biver**

Switzerland

**Jeremy Bland**

UK

**DJ Blizzard**

USA

**Dragica Bobinac**

Croatia

**Christoph Boese**

Germany

**Richard Bohannon**

USA

**Megan Bohensky**

Australia

**Harald Böhm**

Germany

**David Borenstein**

USA

**Pieter Koen Bos**

Netherlands

**Bora Bostan**

Turkey

**Geoff Bove**

USA

**Brendan Boyce**

USA

**Katherine Boyer**

USA

**Elizabeth Bradley**

USA

**Laurence Bradley**

USA

**John Brandsema**

USA

**Ann Bremander**

Sweden

**Sharon Brennan-Olsen**

Australia

**Michael Briggs**

UK

**Mats Brittberg**

Sweden

**Alexander Brunner**

Switzerland

**Paul Bruno**

Canada

**Adam Bryant**

Australia

**Stefan Buchmann**

Germany

**Benjamin Bücking**

Germany

**Matthew Bucknor**

USA

**Benjamin Buecking**

Germany

**Serena Bugatti**

Italy

**Paul Burgers**

Netherlands

**Ruben Burgos-Vargas**

Mexico

**Sigita Burneikiene**

USA

**Agata Burska**

UK

**Nathalie Busso**

Switzerland

**Mario Cabraja**

Germany

**Henrique Cabrita**

Brazil

**Libertad Cáceres**

Spain

**Jerrilyn Cambron**

USA

**Paul Campbell**

UK

**Stefano Campi**

Italy

**Grant Cannon**

USA

**Patricia Canto**

Mexico

**Barbara Cappelletto**

Italy

**Silvia Capuani**

Italy

**Beatriz Carames Perez**

USA

**S Carbone**

Italy

**Mario Cardiel**

Mexico

**Joshua Carothers**

USA

**Delesha Carpenter**

USA

**Andrew Carr**

UK

**Francesco Carubbi**

Italy

**Christian Carulli**

Italy

**Alan Casey**

Canada

**Matteo Castaldo**

Italy

**Marco Castori**

Italy

**Mark Catley**

Australia

**Luis Catoggio**

Argentina

**Nicole Cattano**

USA

**Fulvia Ceccarelli**

Italy

**Luis Cerezal**

Spain

**Uphar Chamoli**

Australia

**BP Chan**

Hong Kong

**Cliffton Chan**

Australia

**Kinwei Chan**

Taiwan

**Eric Chang**

USA

**Connie Chang**

USA

**Eric Chang**

USA

**Moon Jong Chang**

South Korea

**Francisco Javier Chaques**

Spain

**Caroline Chebli**

USA

**Chun Chen**

China

**Huajiang Chen**

China

**Tony Chen**

USA

**Yen-Jen Chen**

Taiwan

**Zhaoyu Chen**

China

**L Cheng**

China

**Jeffrey Cherian**

USA

**Louis Cheung**

Hong Kong

**Wing-Hoi Cheung**

Hong Kong

**Alessandro Chiarotto**

Netherlands

**Philip Chilibeck**

Canada

**Maria Sole Chimenti**

Italy

**Woojin Cho**

USA

**Hyon Choi**

USA

**William Chong**

USA

**Dean Chou**

USA

**James Chow**

USA

**Cara Cipriano**

USA

**Emma Clark**

UK

**Bart Clarke**

USA

**Daniel Cleather**

UK

**Peter Clegg**

UK

**Gerald Coakley**

UK

**Laura Coates**

UK

**Philip Cohen**

USA

**Stephen Coleman**

USA

**Natalie Collins**

Australia

**James Cone**

USA

**Richard Conway**

Ireland

**Chad Cook**

USA

**Andrew Cooke**

Canada

**Brooke Coombes**

Australia

**Oscar J Cordero**

Spain

**Stephen Cornish**

Canada

**Pascal Cosette**

France

**Nigel Cox**

UK

**Jack Crosbie**

Australia

**Maria Cubria**

USA

**Magali Cucchiarini**

Germany

**Antonio Ignacio Cuesta-Vargas**

Spain

**Yong Cui**

China

**Douglas Curran-Everett**

USA

**Olga Cvijanovic**

Croatia

**Dorothea Daentzer**

Germany

**Scott Daffner**

USA

**Chitra Dahia**

USA

**Luis Fernando Dahmer Peruchini**

Brazil

**Zoe Dailiana**

Greece

**Peter Daley**

Canada

**Michael Dan**

Australia

**Abhijeet Danve**

USA

**Amile Dario**

Australia

**Jose Antonio Da Silva**

Portugal

**Fereydoun Davatchi**

Iran

**Aileen Davis**

Canada

**John Davis**

USA

**Edward Dawe**

UK

**Isabel De Salis**

UK

**Nienke De Vries**

Netherlands

**Elizabeth Dean**

Canada

**Benjamin Dean**

UK

**Carlo Felice De Biase**

Italy

**Diederik De Cock**

Belgium

**David Deehan**

UK

**Massimo De Filippo**

Italy

**Jesús Delgado Calle**

Spain

**Annalisa Del Prete**

Italy

**Luis Del Rio**

Spain

**Marco Demange**

Brazil

**C Deml**

Austria

**Craig Denegar**

USA

**Hao Deng**

China

**Elaine Dennison**

UK

**Michael Deschenes**

USA

**Francois Desmeules**

Canada

**Jacqueline Detert**

Germany

**James Devocht**

USA

**Anak Dharmapatni**

Australia

**Arjun Dhawale**

India

**Thomas Dienstknecht**

Germany

**Alberto Diez**

Spain

**Alexandra Dima**

Netherlands

**Charles Dinarello**

USA

**Flavia Di Pietro**

Australia

**Konstantina Dipla**

Greece

**Sara Dockrell**

Ireland

**Dennis Dolny**

USA

**Julio Domenech**

Spain

**Davide Maria Donati**

Italy

**Jian Dong**

China

**Sergey Dorozhkin**

Russian Federation

**Aron Downie**

Australia

**Jeffrey Driban**

USA

**Georgios Drosos**

Greece

**Wei Du**

China

**Arnaud Dubory**

France

**Luzi Dubs**

Switzerland

**Stefan Dudli**

Switzerland

**Alyssa Dufour**

USA

**Sampat Dumbre Patil**

India

**Fiona Duncan**

UK

**Felix Dyrna Md**

USA

**Jennifer Earl-Boehm**

USA

**P. Scott Eastman**

USA

**Benny Samuel Eathakkattu Antony**

Australia

**N. Lawrence Edwards**

USA

**Safak Ekinci**

Turkey

**Pierre Eleniste**

USA

**Maha El Gaafary**

Egypt

**Sherif El Ghazaly**

Egypt

**Andrew W. Eller**

USA

**Yasser El Miedany**

UK

**Sameh Elmorsy**

Japan

**Mahmoud El-Rosasy**

Egypt

**Michaela Endres**

Germany

**Carsten Englert**

Germany

**Christina Engstrand**

Sweden

**Antti Eskelinen**

Finland

**Sean Esmende**

USA

**Emmanuel Estrella**

Philippines

**Max Ettinger**

Germany

**Roni Evans**

USA

**Nicola Fairhall**

Australia

**Johannes Fakler**

Germany

**Miguel Farfán**

Colombia

**Joshua Farr**

USA

**Jonathan Farthing**

Canada

**C Fary**

Australia

**Marta Favero**

Italy

**Qi Fei**

China

**David Feinstein**

USA

**Reed Ferber**

Canada

**Robert Ferrari**

Canada

**Silvano Ferrari**

Italy

**Manuela Ferreira**

Australia

**Jonathan Field**

UK

**Danilo Fintini**

Italy

**John Fitzgerald**

USA

**Martin Fleck**

Germany

**Tapio Flinkkilä**

Finland

**Pawel Flont**

Poland

**Margaret Woon Man Fok**

Hong Kong

**Samo Fokter**

Slovenia

**Nancy Forget**

Canada

**Philipp Forkel**

Germany

**Nicholas Robert Forsyth**

UK

**Matteo Fosco**

Italy

**Calogero Foti**

Italy

**Andreas Fottner**

Germany

**Andrew Franklyn-Miller**

Australia

**Patrick Fransen**

Belgium

**Michael Freeman**

USA

**Julien Freitag**

Australia

**Helen French**

Ireland

**Simon French**

Canada

**Arno Frigg**

Switzerland

**Ts Fu**

Taiwan

**Eisaku Fujimoto**

Japan

**Kanji Fukuda**

Japan

**Naoshi Fukui**

Japan

**Bridgette Furman**

USA

**Takefumi Furuya**

Japan

**André Gächter**

Switzerland

**Angelo Gaffo**

USA

**Jacek Gagala**

Poland

**James Gaida**

Australia

**Sean Gallagher**

USA

**Jiri Gallo**

Czech Republic

**James Galloway**

UK

**Ek Gamstedt**

Sweden

**Rajiv Gandhi**

Canada

**Benjamin Gantenbein**

Switzerland

**Songtao Gao**

China

**Yanzheng Gao**

China

**Juan Garbalosa**

USA

**Serafin Garcia-Mata**

Spain

**Pietro Garofalo**

Italy

**Patel Gayatri**

India

**Jennifer Geddes-McAlister**

Canada

**Sebastian Gehmert**

Switzerland

**Sophie Georgin-Lavialle**

France

**Bjorn Gerdle**

Sweden

**Habib Ghasemi**

Denmark

**Mohammad Ghorbanhoseini**

USA

**Claudia Giacomozzi**

Italy

**Johannes Giesinger**

Netherlands

**Bernadette Gillick**

USA

**Federica Ginanneschi**

Italy

**Vincenzo Giordano**

Brazil

**J Girard**

France

**Melita Giummarra**

Australia

**Cees Glas**

Netherlands

**Ronald Glick**

USA

**Atul Goel**

India

**Paritosh Gogna**

India

**Judith Gold**

Sweden

**Yvonne Golightly**

USA

**Pedro Augusto Gondim Teixeira**

France

**Rachael Gooberman-Hill**

UK

**Taco Gosens**

Netherlands

**Jerzy Gosk**

Poland

**Richard Gouron**

France

**Gertraud Gradl**

Germany

**Jan Grassmann**

Germany

**Ed Greenfield**

USA

**James Greenwood**

UK

**Phillip Gribble**

USA

**Xavier Griffin**

UK

**Tim Griffin**

USA

**Brunella Grigolo**

Italy

**Ram Gudavalli**

USA

**Enrique Guerado**

Spain

**Paulo Guerra**

Brazil

**Sasha Gulati**

Norway

**Jianping Guo**

China

**Shigong Guo**

UK

**Zhiling Guo**

USA

**Prateek Gupta**

USA

**Carl Haasper**

Germany

**Kaken Habaxi**

China

**Sandra Häberle**

Germany

**Fares S. Haddad**

UK

**Ellen Merete Hagen**

Norway

**Yoshiyuki Hakeda**

Japan

**Alan Hakim**

USA

**Michiyuki Hakozaki**

Japan

**Michelle Hall**

Australia

**Keith Hamel**

USA

**Sommer Hammoud**

USA

**Mark Hamrick**

USA

**Kamel Hamzaoui**

Tunisia

**Mark Hancock**

Australia

**Helen Handoll**

UK

**Matthew Hannon**

USA

**Tb Hansen**

Denmark

**Tony Hansson**

Sweden

**Hideaki Hara**

Japan

**Timothy Hardingham**

UK

**Rowan Hardy**

Australia

**Liora Harel**

Israel

**Leila Harhaus**

Germany

**Melissa Harris**

Australia

**Mark Harrison**

Canada

**Robert Hartman**

USA

**Frank Hartmann**

Germany

**Jan Hartvigsen**

Denmark

**Yamada Harumoto**

Japan

**Masahiro Hasegawa**

Japan

**Robin Haskins**

Australia

**Gillian Hatfield**

Canada

**Ellen-Margrethe Hauge**

Denmark

**Tammy Haut Donahue**

USA

**Daniël Haverkamp**

Netherlands

**Eric Hay**

France

**Daichi Hayashi**

USA

**David Haynes**

Australia

**Baorong He**

China

**Emma Healey**

UK

**Bryan Heard**

Canada

**Sajan Hegde**

India

**Lars Helbig**

Germany

**Michael Hellman**

USA

**Yves Henchoz**

Switzerland

**Dirk Henrich**

Germany

**A Nders Henricson**

Sweden

**Yves Henrotin**

Belgium

**Nicholas Henschke**

Germany

**Daniel Hensler**

Germany

**Daniel Hernandez-Vaquero**

Spain

**Philippe Hernigou**

France

**Manfred Herold**

Austria

**Fernando Herrera**

USA

**Gabriel Herrero-Beaumont**

Spain

**Lise Hestbaek**

Denmark

**Christoph- E. Heyde**

Germany

**John Highton**

New Zealand

**Jonathan Hill**

UK

**Stephanie Hinse**

Australia

**Koji Hiraoka**

Japan

**Michael Hirschmann**

Switzerland

**Akihiko Hiyama**

Japan

**Carmen Ho**

Hong Kong

**Maik Hoberg**

Germany

**W Hogge**

USA

**Lisa Hoglund**

USA

**Zhang Hong**

USA

**Thomas Hoogeboom**

Netherlands

**Davyd R. Hooper**

Canada

**Gary Hooper**

New Zealand

**Sven Hoppe**

Switzerland

**Keisuke Horiuchi**

Japan

**Ian Horsley**

UK

**Yuichi Hoshino**

Japan

**M Hösl**

Germany

**Toru Hosoi**

Japan

**Saiyun Hou**

USA

**Lin-Fen Hsieh**

Taiwan

**Ru-Lan Hsieh**

Taiwan

**Jason Hsu**

USA

**Yi Hua**

USA

**Da-Geng Huang**

China

**Hsiaomin Huang**

USA

**Gail Huber**

USA

**Erica Huber**

Switzerland

**Markus Hübscher**

Australia

**John Hughes**

UK

**Carel Hulshof**

Netherlands

**Patricia Hunter**

UK

**Sheree Hurn**

Australia

**Kellie Huxel Bliven**

USA

**Sharon Hyzy**

USA

**Christoph Ihle**

Germany

**Thomas Ilchmann**

Switzerland

**Maria Inacio**

Australia

**Nevsun Inanc**

Turkey

**Pier Francesco Indelli**

USA

**Mm Innmann**

Germany

**Susumu Inoue**

USA

**H Inui**

Japan

**Ulrich Irlenbusch**

Germany

**Kazunari Ishida**

Japan

**Muneaki Ishijima**

Japan

**Yuyu Ishimoto**

Japan

**Tomotaka Ito**

Japan

**Norimasa Iwasaki**

Japan

**Jagnoor Jagnoor**

Australia

**Babak Jahromi**

USA

**Sanjeev Jain**

India

**Nitin Jain**

USA

**Catherine Jankowski**

USA

**Hendrik Jansen**

Germany

**Jacob Jaremko**

Canada

**Mohamed Jarraya**

USA

**David Jayne**

UK

**Ole Kudsk Jensen**

Denmark

**Tue Secher Jensen**

Denmark

**Dae-Geun Jeon**

South Korea

**Jian-Yuan Jiang**

China

**Lei Jiang**

China

**Nan Jiang**

China

**Qing Jiang**

China

**Kitti Jirarattanaphochai**

Thailand

**Lara Joaquín**

Chile

**Beverly Johnson**

USA

**Bruce Jones**

USA

**Elena Jones**

UK

**Andrew Judge**

UK

**Shanil Juma**

USA

**Takahashi Jun**

Japan

**Jesse Jupiter**

USA

**Amir RezaKachooei**

Iran

**Hiroshi Kaji**

Japan

**George Kalliolias**

USA

**Sadishkumar Kamalanathan**

India

**Steven Kamper**

Australia

**Rita Kandel**

Canada

**Yuko Kaneko**

Japan

**Shozo Kanezaki**

Japan

**James Kang**

USA

**Bhaveen Kapadia**

USA

**Christian Kaps**

Germany

**Saffet Karaca**

Turkey

**Feyza Karagöz Güzey**

Turkey

**Joseph Kardouni**

USA

**Yasmaine Karel**

Netherlands

**Afshin Karimi**

USA

**Jaro Karppinen**

Finland

**Morten Karsdal**

Denmark

**Philip Kasten**

Germany

**Susan Kattapuram**

USA

**Jeffrey Katz**

USA

**Hoda Kavosi**

Iran

**Hiroshi Kawaguchi**

Japan

**Yohei Kawakami**

Japan

**Seiji Kawano**

Japan

**Takayuki Kawasaki**

Japan

**Altaf Ahmad Kawoosa**

India

**Burcin Kececi**

Turkey

**Helen Keen**

Australia

**Joern Kekow**

Germany

**Joanne Kemp**

Australia

**Justin Keogh**

Australia

**J. Christiaan Keurentjes**

Netherlands

**Tahir Khan**

UK

**Majed Khraishi**

Canada

**Piya Kiatisevi**

Thailand

**Dana Kidder**

UK

**Jin-Sung Kim**

South Korea

**Sae Hoon Kim**

South Korea

**Sung Kyu Kim**

South Korea

**A Kimura**

Japan

**Atsushi Kimura**

Japan

**Yuka Kimura**

Japan

**T Kirchgesner**

Belgium

**Chlodwig Kirchhoff**

Germany

**Serafim Kiriakidis**

UK

**Andrew Kittelson**

USA

**Christoph Kittl**

Austria

**Heidi Klakk**

Denmark

**Andreas Klatt**

Germany

**Alison Klika**

USA

**Matthias C.M. Klotz**

Germany

**Bas Kluitenberg**

Netherlands

**M Knobe**

Germany

**Patrick Knott**

USA

**Jeffrey Knox**

USA

**Hiroshi Kobayashi**

Japan

**Takashi Kobayashi**

Japan

**Hakan Kocaoglu**

Turkey

**M Koda**

Japan

**Izaäk Kodde**

Netherlands

**Cheryl Koehn**

Canada

**Daisuke Koga**

Japan

**Hideyuki Koga**

Japan

**In Jun Koh**

South Korea

**Zinon Kokkalis**

Greece

**Werner Kolb**

Germany

**Elizaveta Kon**

Italy

**Alice Kongsted**

Denmark

**Kika Konstantinou**

UK

**Evangelos Kontopantelis**

UK

**Kevin Koo**

Singapore

**Sebastian Kopf**

Germany

**Panagiotis Korovessis**

Greece

**Tanja Kostuj**

Germany

**Alpesh Kothari**

UK

**Mark Kotowicz**

Australia

**Eiki Koyama**

USA

**Leyla Kozaci**

Turkey

**Agnessa Kozak**

Germany

**Franz Kralinger**

Austria

**Sandra Kraljevic Pavelic**

Croatia

**Roman Krawetz**

Canada

**Wally Krengel**

USA

**Martin Kretzschmar**

Switzerland

**Morten Tange Kristensen**

Denmark

**Hollis Krug**

USA

**Jan Kuiper**

USA

**Kenji Kumagai**

Japan

**Malhar Kumar**

India

**Yogesh Kumar**

USA

**Steve Kurtz**

USA

**Linda Kwakkenbos**

Canada

**Oh-Yun Kwon**

South Korea

**Pauline Siew Mei Lai**

Malaysia

**Sandra Lakke**

Netherlands

**Polly Lama**

USA

**José Lamo-Espinosa**

Spain

**Kerstin Landin-Wilhelmsen**

Sweden

**Jonathan Larkin**

USA

**Laura Laslett**

Australia

**Richard Lass**

Austria

**Aisha Lateef**

Singapore

**Bruce Latimer**

USA

**Anne Laulund**

Denmark

**Dana Lawrence**

USA

**Andrew Leaver**

Australia

**Byung Ho Lee**

South Korea

**Dave Lee**

Singapore

**Gun Woo Lee**

South Korea

**Hopin Lee**

Australia

**Jeff Leiter**

Canada

**Willem Lems**

Netherlands

**Andreas Leonidou**

UK

**Panagiotis Lepetsos**

Greece

**Lindsey Lepley**

USA

**Gemma Lepri**

Italy

**Andre Leumann**

Switzerland

**Ying Ying Leung**

Singapore

**Brett Levine**

USA

**Alyn Lewis**

UK

**Kirsten Leyland**

UK

**Ang Li**

USA

**Chengliu Li**

USA

**Hongshuai Li**

USA

**Kuan-Yi Li**

Taiwan

**Jing Li**

China

**Quan-Zhen Li**

USA

**Xudong Li**

USA

**Yong-Qi Li**

USA

**Michael Christian Liebensteiner**

Austria

**Dennis Liem**

Germany

**Seung-Jae Lim**

South Korea

**Seung-Jae Lim**

South Korea

**Rong Limin**

China

**Jinn Lin**

Taiwan

**Cheng-Feng Lin**

Taiwan

**Tzu-Hua Lin**

USA

**Liow Lincoln**

Singapore

**Emily Lindley**

USA

**Rene Lindstrøm**

Denmark

**David R Lionberger**

USA

**Sabine Lippacher**

Germany

**Sebastian Lippross**

Germany

**Gina Lisignoli**

Italy

**Chris Littlewood**

UK

**Jiaming Liu**

China

**Jun Liu**

China

**Junting Liu**

China

**Liehua Liu**

China

**R Liu**

USA

**Shaoyu Liu**

China

**Lisa Lix**

Canada

**Margery Lockard**

USA

**Alayna Loiselle**

USA

**Gloria Lopez**

Spain

**Mattia Loppini**

Italy

**JK Lord**

USA

**John Loughlin**

UK

**Quinette Louw**

South Africa

**Anna Lowe**

UK

**Linchao Lu**

USA

**Xuhua Lu**

China

**Yu Lu**

China

**Gregory Lubiani**

USA

**Brittney Luc**

USA

**Juan Luciano**

Spain

**Nada Lukkahatai**

USA

**Teija Lund**

Finland

**Jiaquan Luo**

China

**Hannu Luomajoki**

Switzerland

**Alejandro Luque-Suarez**

Spain

**Shai Luria**

Israel

**Frank Luyten**

Belgium

**Shaw-Ruey Lyu**

Taiwan

**Luciana Macedo**

Canada

**Gustavo Machado**

Australia

**George Macheras**

Greece

**Martin Mackey**

Australia

**Fiona MacKichan**

UK

**Shingo Maeda**

Japan

**Nicola Maffulli**

UK

**Costis Maganaris**

UK

**Asmaa Mahmoud Ali Moustafa**

Egypt

**Edyta Majorczyk**

Poland

**Sharmila Majumdar**

USA

**Kin Cheung Mak**

Hong Kong

**Charles Malemud**

USA

**Rajesh Malhotra**

India

**Theodore Malinin**

USA

**Krishnamurthy Saraswathi Manjunath**

India

**Sithombo Maqungo**

South Africa

**Meir Marmor**

USA

**Antonio Marmotti**

Italy

**Hubert Marotte**

France

**Elisa Marques**

Portugal

**Frank Martetschläger**

Germany

**Janne Martikainen**

Finland

**Nicolo Martinelli**

Italy

**Daniel Martinez-Laguna**

Spain

**M Martin-Ferrero**

Spain

**Youssef Masharawi**

Israel

**Stefano Masiero**

Italy

**Aidin Masoudi**

USA

**Tahir Masud**

UK

**Yuichiro Matsui**

Japan

**Glenn Matsushima**

USA

**Takehiko Matsushita**

Japan

**Tokio Matsuzaki**

Japan

**Stefano Mattioli**

Italy

**G Matziolis**

Germany

**Andreas Mavrogenis**

Greece

**Paolo Mazzola**

Italy

**Chidozie Mbada**

Nigeria

**Jenny McConnell**

Australia

**Daniel McCulloch**

Australia

**Catriona McDaid**

UK

**Suzanne McDonough**

USA

**Meghan McGee-Lawrence**

USA

**Tracy McGregor**

USA

**Neil McHugh**

UK

**C. Wayne McIlwraith**

USA

**Aideen McInerney-Leo**

Australia

**Eileen McNeely**

USA

**Amy McNulty**

USA

**Emily McWalter**

USA

**Daniel McWilliams**

UK

**Damian Medici**

USA

**Geert Meermans**

Belgium

**Christian Meier**

Switzerland

**Marrigje Meijer**

Netherlands

**Riccardo Meliconi**

Italy

**Stephen Mellon**

UK

**Ehud Mendel**

USA

**Christopher Mendias**

USA

**Matthew Menon**

Canada

**Demissew Mern**

Austria

**Addisu Mesfin**

USA

**Karen Messing**

Canada

**James Michelson**

USA

**Toshimi Michigami**

Japan

**Michael Mienaltowski**

USA

**Paola Migliorini**

Italy

**Salvatore Minnella**

Italy

**Frederico Miranda**

Brazil

**Smarak Mishra**

UK

**Thomas Mittlmeier**

Germany

**Junichi Miyake**

Japan

**Takeshi Miyamoto**

Japan

**Ali Mobasheri**

UK

**Maarten Moen**

Netherlands

**Omar Mohamed**

Germany

**Mohamed Mohi Eldin**

Egypt

**Rahim Moineddin**

Canada

**Ingrid Moller**

Spain

**Halima Moncrieffe**

USA

**Robert Moots**

UK

**B Moradi**

Germany

**Jaime Morales**

Spain

**Kanji Mori**

Japan

**Fintan Moriarty**

Switzerland

**Suzanne Morin**

Canada

**Akio Morinobu**

Japan

**Toru Moro**

Japan

**Rohan Morris**

UK

**Seyed Javad Mousavi**

Australia

**Sara Muller**

UK

**Marcus Mumme**

Switzerland

**Shigeyuki Muraki**

Japan

**Tsuyoshi Murase**

Japan

**George Murrell**

Australia

**Harun Mutlu**

Turkey

**Es Mwaka**

Uganda

**Corrie Myburgh**

Denmark

**Keiji Nagata**

Japan

**Istvan Nagy**

UK

**Bindu Nair**

Canada

**Yusuke Nakagawa**

Japan

**Atsuo Nakamae**

Japan

**Naoki Nakano**

Japan

**Tomoyuki Nakasa**

Japan

**Arvind Nana**

USA

**Kutty Selva Nandakumar**

Sweden

**Michael Nasr**

USA

**Nagwa Nassar**

Egypt

**Jose Luis Navarro-Espigares**

Spain

**Sangeetha Naveen**

Malaysia

**Justine Naylor**

Australia

**Ara Nazarian**

USA

**Mwidimi Ndosi**

UK

**Subas Neupane**

Finland

**D Newell**

UK

**Johnathan Ng**

USA

**Leo Ng**

Australia

**Leslie Nicholson**

Australia

**Andreas Niemeier**

Germany

**Thomas Niethammer**

Germany

**Pieter Nieuwenhuijzen**

Netherlands

**Svetla Nikolova**

Bulgaria

**Jun Nishida**

Japan

**Yuichiro Nishizawa**

USA

**Daniele Noel**

France

**Marcello Nogueira-Barbosa**

Brazil

**JH Noh South**

Korea

**Peter Norman**

UK

**Andrej Nowakowski**

Switzerland

**Georgia Ntani**

UK

**Mary Nugent**

Ireland

**Michael Nunns**

UK

**Alison Oates**

Canada

**Neil O'Connell**

UK

**Pat O'Connor**

USA

**David O'Gorman**

Canada

**Britt Elin Øiestad**

Norway

**Mary O'Keeffe**

Ireland

**Francesco Oliva**

Italy

**Rene Olivares-Navarrete**

USA

**Chris Oliver**

UK

**Gunnar Olsson**

Sweden

**Go Omori**

Japan

**Patrick Omoumi**

Switzerland

**Raymond Oppong**

UK

**Ana-Maria Orbai**

USA

**Viveca Östberg**

Sweden

**Monika Ostensen**

Norway

**Georg Osterhoff**

Switzerland

**Kieran O'Sullivan**

Ireland

**Leonard O'Sullivan**

Ireland

**Takemi Otsuki**

Japan

**Merry Oursler**

USA

**Simon Øverland**

Norway

**Levent Ozcakar**

Turkey

**Guven Ozkaya**

Turkey

**Keiichi Ozono**

Japan

**Yusuf Öztürkmen**

Turkey

**Patrick Pabian**

USA

**Verity Pacey**

Australia

**Jukka Pajarinen**

USA

**Paolo Paladini**

Italy

**Shea Palmer**

UK

**Thorvaldur Palsson**

Denmark

**Wen Pan**

USA

**Danilo Pani**

Italy

**Ranajit Panigrahi**

India

**Evangelos Pappas**

Australia

**John Pappas**

USA

**Eric Parent**

Canada

**Jang Won Park**

South Korea

**Jin KyunPark**

South Korea

**Won Sang Park**

South Korea

**Martyn Parker**

UK

**Sharon Parry**

Australia

**Zoe Paskins**

UK

**Debabrata Patra**

USA

**Jochen Paul**

Germany

**Elena Pavlova**

UK

**Imre Pavo**

Austria

**Mark Pearson**

UK

**Ashley Pedler**

Australia

**Nancye May Peel**

Australia

**Fuxing Pei**

China

**Zhang Peixun**

China

**Leonardo Pellicciari**

Italy

**Baogan Peng**

China

**Erika Penz**

Canada

**Anneli Peolsson**

Sweden

**Melanie Pepin**

USA

**Beatriz Pérez-Zafrilla**

Spain

**Harris Perlman**

USA

**Yves-Marie Pers**

France

**Ákos Pethes**

Hungary

**Susan Picavet**

Netherlands

**Motta Pierorazio**

Italy

**Patrícia Pinto**

Portugal

**Rafael Pinto**

Australia

**Spiros Pneumaticos**

Greece

**Denis Poddubnyy**

Germany

**Jonas Pogorzelski**

Germany

**Brian Poole**

USA

**Hadi Poormoghim**

Iran

**Janet Pope**

Canada

**Mark Porcheret**

UK

**Alexey Porollo**

USA

**Fereshteh Pourkazemi**

Australia

**James Prior**

UK

**Ashleigh Prowse**

Australia

**Thomas Pufe**

Germany

**Paul Purdue**

USA

**Zhao Qing Hua**

China

**Gerald Quan**

Australia

**Danuta Radzioch**

Canada

**Stefan Rahm**

Switzerland

**Muhammad Farooq Rai**

USA

**Yolande F. M. Ramos**

Netherlands

**Francesco Ranuccio**

Italy

**Lasse Enkeboelle Rasmussen**

Denmark

**Eva Rasmussen-Barr**

Sweden

**Akhilesh Rathi**

India

**Michael Skovdal Rathleff**

Denmark

**Trishna Rathod**

UK

**Frank Rauch**

Canada

**Jennifer Read**

UK

**Michael Reade**

Australia

**Trudy Rebbeck**

Australia

**Sakamuri Reddy**

USA

**Martine Regenboog**

Netherlands

**Thomas Reichel**

Germany

**Duncan Reid**

New Zealand

**Monique Reijnierse**

Netherlands

**Olav Reikerås**

Norway

**Michael Reiman**

USA

**Jens Reimann**

Germany

**Dajiang Ren**

China

**Xiang Ren**

China

**Babst Reto**

Switzerland

**Jose A. Riancho**

Spain

**Marcelo Riberto**

Brazil

**Jim Richards**

UK

**Robin Richards**

USA

**James B Richardson**

UK

**Julie Richardson**

Canada

**Jane Richardson**

UK

**Jochen Ringe**

Germany

**Bernhard Rintelen**

Austria

**Fernando Rivadeneira**

Netherlands

**Shawn Robbins**

Canada

**Timothy Roberts**

USA

**Philip Robinson**

Australia

**Rita Rocha**

Portugal

**Scott Rodeo**

USA

**Götz Röderer**

Germany

**Vitor Rodrigues**

Portugal

**Sattaya Rojanasthien**

Thailand

**Tobias Romeyke**

Austria

**Keith Rose**

USA

**Joseph Rosen**

USA

**Dieter Rosenbaum**

Germany

**Ann Rosenthal**

USA

**Barbara Rossi**

Italy

**Roberto Rossi**

Italy

**Graciela Rovner**

Sweden

**Flynn Rowan**

USA

**Paula Rozenfeld**

Argentina

**Dike Ruan**

China

**L. Ruggiero**

Italy

**Cormac Ryan**

UK

**Joachim Rychly**

Germany

**Luigi Sabatini**

Italy

**Ahmed Sadek**

Egypt

**Patrick Sadoghi**

Austria

**Surachai Sae-Jung**

Thailand

**Jayaprakash Sahoo**

India

**Kulvinder Singh Saini**

Saudi Arabia

**Taku Saito**

Japan

**Masayuki Saka**

Japan

**Takashi Sakai**

Japan

**Akio Sakamoto**

Japan

**Km Salem**

UK

**Donald Salter**

UK

**John Salvo**

USA

**Afshin Samani**

Denmark

**Nemandra Sandiford**

UK

**Gunther H. Sandmann**

Germany

**Bruno Saragiotto**

Australia

**Joseph Sarver**

USA

**Vishal Sarwahi**

USA

**Hiroshi Sasaki**

USA

**Ben Saunders**

UK

**Judith Sautner**

Austria

**Olga Savvidou**

Greece

**Tetsuji Sawada**

Japan

**Frederieke Schaafsma**

Australia

**Ellen Scherl**

USA

**Philip Scheurer**

Netherlands

**Aaron Schindeler**

Australia

**Ulf Jens Schlegel**

Germany

**Anneliese Schleyer**

USA

**Benedikt Schliemann**

Germany

**Tanja Schmidt**

Germany

**Tannin Schmidt**

Canada

**J Schmolders**

Germany

**Michael Schnabel**

Germany

**Michael Schneider**

USA

**Arndt P. Schulz**

Germany

**Gundula Schulze-Tanzil**

Germany

**Philipp Schwabe**

Germany

**Cedric Schwartz**

Belgium

**Edward Schwarz**

USA

**Ran Schwarzkopf**

USA

**Richard Schwend**

USA

**Jason Scibek**

USA

**John Scolaro**

USA

**David Scott**

UK

**Whitney Scott**

UK

**Anna Scotto D'Abusco**

Italy

**Mamdouh Sedhom**

Australia

**Leyla Sedighipour**

USA

**Miho Sekiguchi**

Japan

**Jonathan Sembrano**

USA

**Emily Seto**

Canada

**F Sevelda**

Austria

**Deepak Sharan**

India

**William Shaw**

USA

**Charles Sheets**

USA

**Julia Shelton**

UK

**Demissew Shenegelegn Mern**

Austria

**Ajoy Prasad Shetty**

India

**Yusuke Shinoda**

Japan

**Masataka Shiraki**

Japan

**Debra Shirley**

Australia

**Yehuda Shoenfeld**

Israel

**AJ Shyam Kumar**

UK

**Marcin Sibinski**

Poland

**Nektaria Sidiropoulou**

Greece

**Siddhartha Sikdar**

USA

**Milena Simic**

Australia

**Jasvinder Singh**

USA

**Sanna Sinikallio**

Finland

**Juha-Jaakko Sinikumpu**

Finland

**Francisca Sivera**

Spain

**Susan Slade**

Australia

**David Slattery**

Australia

**Gerard Slobogean**

USA

**Francesco Smeraglia**

Italy

**Theodoor H. Smit**

Netherlands

**Jo Smith**

UK

**Lachlan Smith**

USA

**Tuulikki Sokka**

Finland

**Leonid Solomin**

Russian Federation

**Matthijs Somford**

Netherlands

**Enrique R. Soriano**

Argentina

**Miguel Sousa**

Portugal

**Richard Souza**

USA

**Dusko Spasovski**

Serbia

**Mauro Spina**

Italy

**Christian Spross**

Switzerland

**Sander Spruijt**

Netherlands

**J. Bart Staal**

Netherlands

**D. Stacey**

Canada

**Rebecca Stack**

UK

**Daniel Steffens**

Australia

**Ely Steinberg**

Israel

**Arnd Steinbrueck**

Germany

**Dirk Stengel**

Germany

**Joanna Stephen**

UK

**Matthew Stevens**

Australia

**Stefan Stich**

Germany

**Mette Stochkendahl**

Denmark

**Klaus Strobel**

Switzerland

**Sebastian Suarez**

USA

**A Sud**

India

**Tatsuo Suda**

Japan

**Akihiro Sudo**

Japan

**Ruifang Sui**

China

**Walton Sumner**

USA

**Yu Sun**

China

**Björn Sundström**

Sweden

**Wen-Hsu Sung**

Taiwan

**Akari Suzuki**

Japan

**Noboru Suzuki**

Japan

**Tomoyuki Suzuki**

Japan

**Michael Swain**

Australia

**Ganesh Swamy**

Canada

**Grazyna Sypniewska**

Poland

**Uta Syrbe**

Germany

**Pawel Szulc**

France

**Sofi Tagesson**

Sweden

**Mohammad Taghipour Darzi**

Iran

**Yoo Tai June**

USA

**Toshiaki Takahashi**

Japan

**Naoyuki Takahashi**

Japan

**Koji Takayama**

Japan

**Yuji Takazawa**

Japan

**Wen-Hua Tan**

China

**Nobuhiro Tanaka**

Japan

**Veena Taneja**

USA

**Chih-Hsin Tang**

Taiwan

**Noboru Taniguchi**

Japan

**Sarah Tansley**

UK

**Ekamol Tantisattamo**

USA

**Anne-Kathrin Tausche**

Germany

**Rod Taylor**

UK

**Regina Taylor-Gjevre**

Canada

**Andrew Teichtahl**

Australia

**René Ten Broeke**

Netherlands

**Masafumi Terada**

USA

**Masatoshi Teraguchi**

Japan

**Takeshi Teratani**

Japan

**Enrique Adrian Testa**

Switzerland

**Teun Teunis**

Netherlands

**Loukas Thanos**

Greece

**Jean-Claude Theis New**

Zealand

**Brian Theodore**

USA

**Achilleas Thoma**

Canada

**Gethin Thomas**

Australia

**Ellen Thomas**

UK

**Martin Thomas**

UK

**Jeffrey Thomson**

USA

**Andrew Thoreson**

USA

**Stephanie Tien-A-Looi**

USA

**Mark Timmons**

USA

**Thomas Tischer**

Germany

**Derrick Todd**

USA

**Stefan Toegel**

Austria

**Andreas Toepfer**

Germany

**Harukazu Tohyama**

Japan

**Christy Tomkins-Lane**

Canada

**Peijian Tong**

China

**Daniel Tonge**

UK

**Francine Toye**

UK

**Ismail Remzi Tozun**

Turkey

**Marco Traballesi**

Italy

**Adrian Traeger**

Australia

**Vincent Traynelis**

USA

**Lea Trela-Larsen**

UK

**Ilaria Tresoldi**

Italy

**Luisa Trombi**

Italy

**Kimberlee Trudeau**

USA

**Aleksandra Truszczynska**

Poland

**Tsung-Yuan Tsai**

USA

**Elena Tsourdi**

Germany

**Sachiyuki Tsukada**

Japan

**Marcin Tyrakowski**

Poland

**Jan Ulfberg**

Sweden

**J Urrutia**

Chile

**Werner Vach**

Germany

**Guus Van Den Akker**

Netherlands

**Bart Van Den Bemt**

Netherlands

**Peter Van Den Berg**

Netherlands

**Paul Van Den Dolder**

Australia

**Cornelia Van Den Ende**

Netherlands

**Josien Van Den Noort**

Netherlands

**Walter Van Der Weegen**

Netherlands

**Danielle Van Der Windt**

UK

**Peter Van Lent**

Netherlands

**Gerjo Van Osch**

Netherlands

**Ottar Vasseljen**

Norway

**Martti Vastamäki**

Finland

**Patrick Vavken**

USA

**Arianne Verhagen**

Netherlands

**John Verhoef**

Netherlands

**Howard Vernon**

Canada

**Francesca Veronesi**

Italy

**Martin L Verra**

Switzerland

**Suzanne Verstappen**

UK

**E Vialle**

Brazil

**Esther Vicente**

Spain

**Tapio Videman**

Canada

**Edgar Vieira**

USA

**Davide Viggiano**

Italy

**Juan Villa-Camacho**

USA

**Rob Vining**

USA

**Marianna Vlychou**

Greece

**Nam Vo**

USA

**Stephan Vogt**

Germany

**Mauro Volpi**

Brazil

**Philipp Von Roth**

Germany

**Christian Von Rüden**

Germany

**James Vosseller**

USA

**Alessandra Vultaggio**

Italy

**Raimund Wagener**

Germany

**Monique Margaretha Jozefa Walenkamp**

Netherlands

**William Walsh**

Australia

**Nicole Walsh**

Australia

**Edward Wang**

Philippines

**James Wang**

USA

**Jann-Tay Wang**

Taiwan

**Jing Wang**

USA

**Shaobai Wang**

USA

**Yuanyuan Wang**

Australia

**Yum-Tao Wang**

China

**Michael Ward**

USA

**Stuart Warden**

USA

**Fiona Watt**

UK

**Adam Watts**

UK

**M Weber**

Germany

**Fiona Webster**

Canada

**Niels Wedderkopp**

Denmark

**Kilian Wegmann**

Germany

**Lei Wei**

USA

**Conrad Weihl**

USA

**Yoram Weil**

Israel

**Andre Weimann**

Germany

**Harrie Weinans**

Netherlands

**Tomas Weitoft**

Sweden

**Megan Weivoda**

USA

**Chunyi Wen**

Hong Kong

**Maria M. Wertli**

Switzerland

**Michael Whitehouse**

UK

**David Whitehurst**

Canada

**Rod Whiteley**

Qatar

**Harald Widhalm**

Austria

**Karl-Heinz Widmer**

Switzerland

**Susan Wilbanks**

UK

**Amanda Williams**

UK

**Christopher Williams**

Australia

**Valerie Williams**

USA

**Tobias Winkler**

Germany

**John Winslow**

USA

**Patarawan Woratanarat**

Thailand

**Stuart Wright**

UK

**Jau-Ching Wu**

Taiwan

**Guoming Xie**

China

**Jiake Xu**

Australia

**Weidong Xu**

China

**Yue Xuan**

USA

**Meilang Xue**

Australia

**Ryo Yamada**

Japan

**Hisashi Yamanaka**

Japan

**RJ Yan**

China

**Huilin Yang**

China

**Yun-Feng Yang**

China

**Qiuming Yao**

USA

**Hisataka Yasuda**

Japan

**Kensuke Yasumura**

Japan

**Metin Yavuz**

USA

**Xiaodong Yi**

China

**M Yoshida**

Japan

**Hiroyuki Yoshihara**

USA

**Toshitaka Yoshii**

Japan

**Ichiro Yoshimura**

Japan

**Hiroyuki Yoshitomi**

Japan

**Md Yuzaiful Md Yusof**

UK

**Xiuchun Yu**

China

**Hua Yu**

USA

**W Yuan**

China

**Kazuo Yudoh**

Japan

**Liu Yueju**

China

**I Yugué**

Japan

**Andreas Zafeiridis**

Greece

**Fabio Zaina**

Italy

**Ivan Zderic**

USA

**Jian Zhang**

USA

**Jun Zhang**

China

**Kairui Zhang**

China

**Qiang Zhang**

China

**Xiaofan Zhang**

USA

**Xiaojian Zhang**

China

**Ying Zhang**

China

**Fengdong Zhao**

China

**Yong Zhao**

China

**Liying Zheng**

USA

**Fang Zhenhua**

China

**Yongjin Zhou**

China

**Linuo Zhou**

China

**Yuan Zhu**

China

**Ruofu Zhu**

China

**Yi Zhu**

USA

